# Oral toxicity of isotretinoin, misoprostol, methotrexate, mifepristone and levonorgestrel as pregnancy category X medications in female mice

**DOI:** 10.3892/etm.2015.2203

**Published:** 2015-01-22

**Authors:** SEONG-KWAN KIM, SOO-JEONG SHIN, YOHAN YOO, NA-HYUN KIM, DONG-SOON KIM, DAN ZHANG, JIN-A PARK, HEE YI, JIN-SUK KIM, HO-CHUL SHIN

**Affiliations:** 1 Department of Veterinary Pharmacology and Toxicology, College of Veterinary Medicine, Konkuk University, Seoul 143-701, Republic of Korea; 2 Jayang High School, Seoul 143-861, Republic of Korea; 3 Konkuk University High School, Seoul 143-701, Republic of Korea

**Keywords:** toxicity, pregnancy category X drugs, mice

## Abstract

An oral toxicity study of several pregnancy category X drugs was performed in female ICR mice. The drugs were administered orally once daily for 3 days at doses of 1, 10 and 100 μg/kg for isotretinoin; 6.7, 67 and 670 μg/kg for misoprostol; 83, 830 and 8,300 μg/kg for methotrexate; 3.3, 33 and 330 μg/kg for mifepristone; and 25, 250 and 2,500 μg/kg for levonorgestrel. During the test period, clinical signs, mortality, body weight, hematology, serum biochemistry and necropsy findings were examined. Following administration of methotrexate at 8,300 μg/kg, a number of animals exhibited decreased spontaneous activity, and one animal died. In the hematological analysis, compared with those treated with the control, the animals treated with the drugs exhibited similar significant decreases in the number of granulocytes and granulocyte differentiation, and increases in lymphocyte differentiation. In the serum biochemical analysis, animals receiving high doses of the five drugs demonstrated significant changes in uric acid, glucose, alkaline phosphatase, total bilirubin, lipase, total cholesterol and calcium. At necropsy, intestinal redness was frequently observed in animals that received the high dose of methotrexate. Uterus enlargement and ovary dropsy were also detected in the groups receiving mifepristone and levonorgestrel. Despite the short-term exposure, these drugs exhibited significant side effects, including white blood cell toxicity, in the mouse model. Category X drugs can be traded illegally via the internet for the purpose of early pregnancy termination. Thus, illegal abuse of the drugs should be further discouraged to protect mothers.

## Introduction

Numerous teenagers in Korea are under severe social and psychological stresses, particularly due to the extremely competitive system of college admission. Various types of mental conditions, including lack of social skills, violent tendencies, attempted suicide, internet game addiction, smartphone dependence syndrome, smoking, alcohol and drug abuse, and abnormal sexual attitudes and behaviors are emerging as serious social problems. Recently, teenage pregnancy has become an increasing problem (Christian Union Newspaper & Kookmin Ilbo, Korea, 03/17/2010). Pregnancy is considered normal for married couples; however, being an unwed mother at a young age is considered shameful in Korea. In many cases, rather than visit a doctor, young women try to purchase drugs that will induce abortion through internet sites due to their ease of access (KBS, Korea, 12/20/2013). However, such drugs are generally understood to be hazardous compounds due to the severe reproductive damage they cause (i.e., due to their teratogenicity). Therefore, the use of these drugs without a doctor’s prescription is an unsafe practice.

In this study, we have examined several drugs, including isotretinoin, misoprostol, methotrexate, mifepristone and levonorgestrel, that are known to be abortion-inducing compounds. Isotretinoin, an orally active retinoic acid derivative, has been used for the treatment of severe refractory nodulocystic acne for more than 30 years (1,2). Misoprostol, a synthetic prostaglandin E1 methyl ester analog, has potent antisecretory and cytoprotective effects in the treatment of gastric and duodenal ulcers (3,4). Methotrexate has long been used for the treatment of cancers (5). Mifepristone is a progesterone receptor antagonist used as an abortifacient in the early stage of pregnancy and has occasionally been used in the treatment of refractory Cushing’s syndrome (6,7). However, mifepristone is not approved for use in Korea. Levonorgestrel is a second-generation synthetic progestogen used as an active ingredient in certain hormonal contraceptives (8,9). All of these drugs are classified as ‘category X’. Drugs in this category have a high risk of causing permanent damage to the fetus and should not be used during pregnancy. This study was designed to compare the potential toxicity associated with the oral administration of abortion-related compounds for a short time period using ICR mice.

## Materials and methods

### Animal maintenance

Sixty-five female ICR mice (aged 6 weeks) were purchased from Nara Biotech (Seoul, Korea) and were used following a 1-week quarantine and acclimatization period. Four animals per cage were housed in a room maintained at a temperature of 23±3°C with a relative humidity of 50±10%. The animals were provided tap water and commercial rodent chow (NIH-31 Open Formula Auto, Ziegler Bros Inc., Gardners, PA, USA) *ad libitum*. The procedures used in the animal experiment were approved by the Institutional Animal Care and Use Committee, Konkuk University (Seoul, Korea).

### Chemicals

Isotretinoin, misoprostol, methotrexate, mifepristone and levonorgestrel were purchased from Sigma-Aldrich (St. Louis, MO, USA; Table I). The test drugs were dissolved/suspended in 0.75% Tween-80 solution (Sigma-Aldrich), and dosing solutions were prepared daily prior to treatment. The application volume of the drugs was 10 ml/kg body weight and was calculated based on the most recently recorded body weight of each individual animal. The test mixture was administered daily by gavage to female mice for 3 days. Control mice received an equivalent volume of 0.75% Tween-80 solution alone. The oral administration method was selected for this study since these drugs are administered orally in a clinical setting.

### Experimental groups and dose selection

Healthy female mice were randomly assigned to sixteen experimental groups, comprising one control group and fifteen drug treatment groups (4–5 mice per group). Each drug was administered at three doses: low, medium and high. Isotretinoin was administered once daily at doses of 1, 10 and 100 μg/kg; misoprostol was administered at 6.7, 67 and 670 μg/kg; methotrexate was administered at 83, 830 and 8,300 μg/kg; mifepristone was administered at 3.3, 33 and 330 μg/kg; and levonorgestrel was administered at 25, 250 and 2,500 μg/kg. The approximate clinical dose in humans was selected as the low dose, the medium dose was ten times the low dose, and the high dose was ten times the medium dose.

### Clinical observation and mortality

The rats were observed daily for clinical signs as well as physiological and behavioral changes throughout the period of dosing. Toxic manifestations and mortality were also monitored once daily. The body weight of each mouse was measured at the beginning of drug exposure.

### Hematology

The animals were fasted overnight prior to necropsy and blood collection. Blood samples were drawn from the posterior vena cava using a syringe with a 26-gauge needle under ether anesthesia. Blood samples were collected into complete blood count bottles containing EDTA-2 K (Green Cross Medical Industry, Seoul, Korea) and analyzed using an automatic hematology analyzer (Abaxis VetScan^®^ HM2 Hematology System, Union City, CA, USA). The following parameters were determined: red blood cell count, hemoglobin concentration, hematocrit, mean corpuscular volume, mean corpuscular hemoglobin, mean corpuscular hemoglobin concentration, platelet count, total white blood cell (WBC) count, and differential WBC count: lymphocytes, monocytes, granulocytes, percentage of lymphocytes, percentage of monocytes and percentage of granulocytes.

### Serum biochemistry

Blood samples were centrifuged at 3,000 rpm for 10 min within 1 h of collection. The sera were stored at −80°C prior to analysis. The following serum biochemistry parameters were evaluated using an autoanalyzer (Cobas c111 System, Roche Diagnostics Ltd, Rotkreuz, Switzerland): aspartate aminotransferase, alanine aminotransferase, alkaline phosphatase, blood urea nitrogen, creatinine, creatine kinase, lactate dehydrogenase, lipase, glucose, total cholesterol, total bilirubin, total protein, albumin, calcium, inorganic phosphorus and uric acid.

### Gross findings

All surviving animals were anesthetized with ether to collect blood samples at the end of the experiment. The mice were sacrificed by exsanguination from the abdominal aorta. Complete gross postmortem examinations were performed on all terminated animals.

### Statistical analysis

All results are presented as the mean value ± standard deviation (SD). Within-group comparisons were performed using analysis of variance. Significant differences between the control and experimental groups were assessed by Student’s t-test. Gross necropsy findings are represented as frequencies. P<0.05, P<0.01 and P<0.001 were considered to indicate different levels of statistical significance.

## Results

One animal in the high-dose methotrexate (8,300 μg/kg) group was found dead on day 2. The animal did not demonstrate any notable clinical signs prior to death; however, there was a high incidence of decreased activity among the other animals in the high-dose methotrexate group. No abnormal changes in general behavior or other physiological activities were observed in the groups receiving the other drugs.

All animals that survived until necropsy were subjected to hematological examination. As shown in Table II, most of the animals treated with isotretinoin, misoprostol, methotrexate, mifepristone and levonorgestrel demonstrated a significant decrease (P<0.001) in granulocytes count and differentiation percentage compared with the control group. Conversely, lymphocyte differentiation was significantly increased by all drugs with the exception of methotrexate. Misoprostol at a dose of 670 μg/kg/day significantly decreased the total WBC count, and the high dose of mifepristone (330 μg/kg/day) significantly increased the hematocrit level. Certain hematological parameters were also altered by drug treatment; however, a dose-related response was not observed.

The results of the blood biochemical tests are shown in Table III. Compared with the control group, minor changes were observed in the low-dose isotretinoin, misoprostol, methotrexate, mifepristone and levonorgestrel groups. However, various changes related to drug treatment were observed in the medium- and high-dose groups. Isotretinoin caused significant increases in alkaline phosphatase and total bilirubin and decreases in glucose and uric acid at doses of 10 and 100 μg/kg. Misoprostol caused a significant increase in alkaline phosphatase and a reduction in uric acid at a dose of 670 μg/kg. Methotrexate caused a significant increase in lipase and a decrease in uric acid at the high dose of 8,300 μg/kg. Treatment with mifepristone led to a significant increase in total cholesterol and calcium as well as a decrease in glucose at doses of 33 and 330 μg/kg. Levonorgestrel caused a significant increase in calcium and a decrease in glucose at the high dose of 2,500 μg/kg.

**Figure 1:**
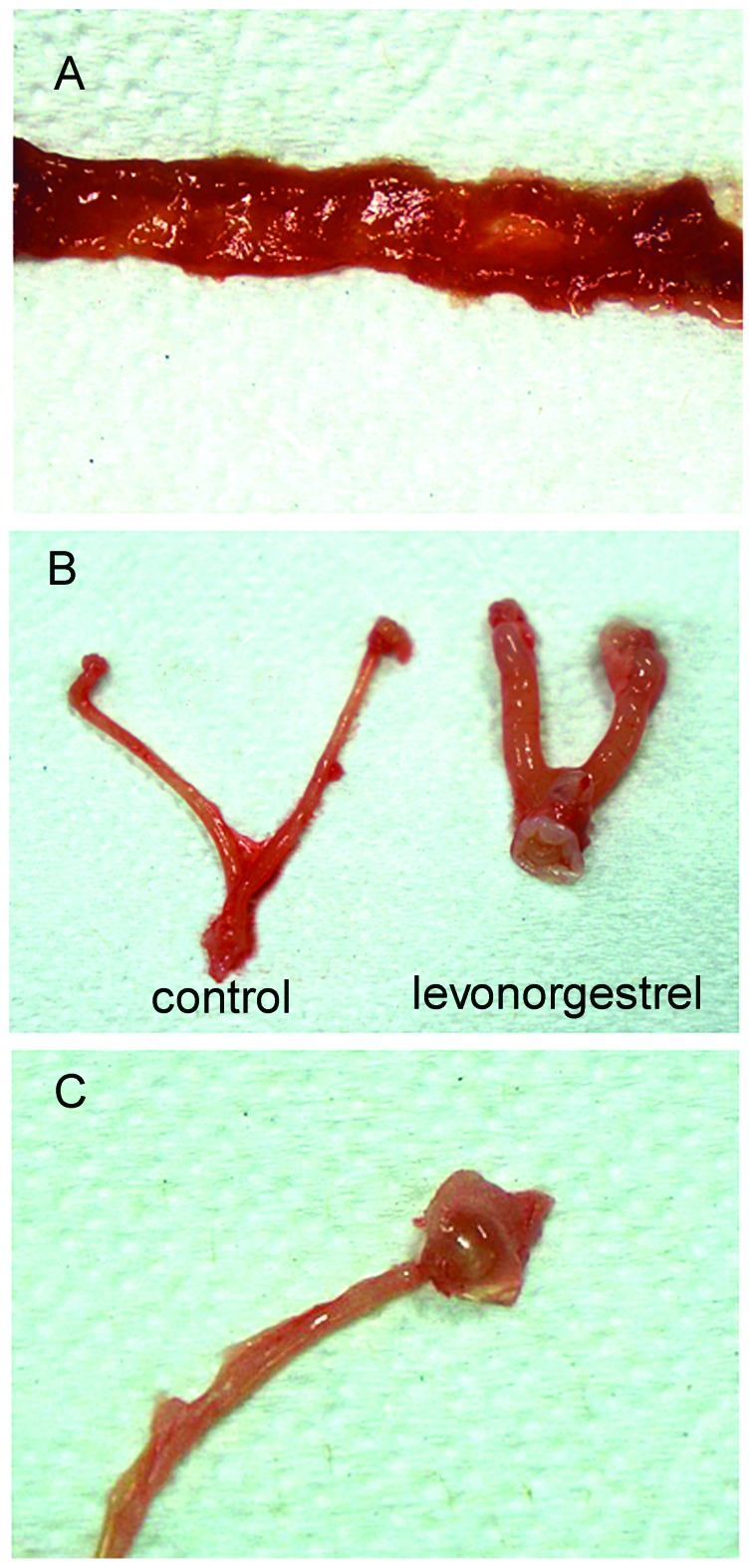
Necropsy findings. (A) Intestine, (B) uterus, (C) ovary.

At the scheduled necropsy, treatment-related gross findings were examined and compared with those of control animals. The single dead animal observed in the high-dose methotrexate (8,300 μg/kg) group exhibited severe gastrointestinal and lung bleeding. As shown in Table IV and Fig. 1, gross changes including intestinal redness, uterine enlargement and ovarian dropsy were detected in the drug treatment groups. Intestinal redness was frequently observed in animals treated with methotrexate at higher doses. Uterine enlargement and ovarian dropsy were repeatedly observed in the mifepristone and levonorgestrel groups.

## Discussion

The present study was conducted to assess the oral toxicity of pregnancy category X drugs, namely isotretinoin, misoprostol, methotrexate, mifepristone and levonorgestrel, in ICR mice. The results of the study revealed that daily oral administration of the drugs for 3 days caused certain changes in the hematological and serum biochemical parameters as well as the gross parameters of organs (based on necropsy findings).

No abnormal clinical signs were observed in any of the animals with the exception of those in the high-dose methotrexate (8,300 μg/kg) group, which demonstrated decreased spontaneous activity. Additionally, there was one dead animal in this group. Methotrexate is a potent antiproliferative and immunosuppressive agent that is widely used against a broad spectrum of cancer-related diseases and arthritis. However, the drug has demonstrated significant toxicity in a number of organs, including the liver, lung, intestine, bone, heart and blood as well as the reproductive and nervous system (10). The LD50 of methotrexate is known to be approximately 50–200 μg/kg in mice.

The hematological system is one of the most sensitive targets for toxic chemicals and is a significant index of physiological and pathological status in humans and animals. In our study, significant changes in the WBC count were observed in the hematological analysis following treatment with category X drugs. All drugs demonstrated similar dose-response correlations, and marked reductions in the numbers of granulocytes and granulocyte differentiation were observed, as well as an increase in the lymphocyte differentiation. These findings indicate that the drugs exhibit hematological toxicity related to WBCs, which are the cells of the immune system that are involved in defending the body against infectious disease and foreign materials. It was reported that isotretinoin treatment induces oxidative toxicity in the blood of patients (11), and several studies have indicated that methotrexate exhibits blood toxicity. Murakami *et al* (12) observed a decreased number of blood cells in rats that were administered low-dose methotrexate. Kose *et al* (13) also reported the severe hematological toxicity of methotrexate. Mifepristone causes severe blood loss due to vaginal bleeding (14–16). Significant reductions in the mean platelet count have been observed due to long-term treatment with levonorgestrel (17).

Based on the serum biochemical analysis, higher doses of the drugs were observed to cause various changes in biochemical parameters including uric acid, glucose, alkaline phosphatase, total bilirubin, lipase, total cholesterol and calcium. Alkaline phosphatase was significantly increased by treatment with isotretinoin and misoprostol, and calcium was increased by mifepristone and levonorgestrel. Total bilirubin, lipase and total cholesterol were also considerably elevated in serum following treatment with isotretinoin, methotrexate and mifepristone. However, the serum glucose level was significantly decreased following treatment with isotretinoin, mifepristone and levonorgestrel. Uric acid was also decreased following treatment with isotretinoin, misoprostol and methotrexate. This result may indicate that the drugs have side effects including hypoglycemic and hypouricemic activity. It has been documented that these drugs exert various effects on serum biochemical parameters. For example, it has been reported that isotretinoin significantly increases the serum levels of aspartate aminotransferase, total cholesterol and triglycerides (18). Another study indicated that oral isotretinoin therapy inhibits bone turnover and calcium homeostasis (19). Elevation of the alkaline phosphatase level in serum was observed in misoprostol-treated rats (20). Ettinger (21) reported a marked increase in uric acid caused by methotrexate treatment. The level of glutamic-oxaloacetic transaminase in serum was also altered following treatment with high-dose methotrexate (22). It was also reported that mifepristone induced severe hypokalemia in cancer patients (23). Other studies have indicated that mifepristone and levonorgestrel are closely associated with calcium and bone metabolism (24,25).

At necropsy, intestinal redness was observed in animals receiving high-dose methotrexate. Indeed, methotrexate-induced intestinal damage in mice has been well documented in earlier studies (26–30). Uterus enlargement and ovary dropsy were frequently detected in the groups receiving mifepristone and levonorgestrel. Although it is not known whether these findings are normal biological variations, Tamura *et al* demonstrated ovarian toxicity in female rats following repeated doses of mifepristone (31).

In conclusion, 3-day repeated oral administration of pregnancy category X drugs to mice resulted in notable changes in WBCs, including a marked reduction of granulocytes and an increase of lymphocytes. Based on the serum analysis, the drugs also caused changes in various biochemical parameters. Therefore, the present study suggests that these drugs may induce blood toxicity in mice despite the short-term exposure. Thus, it is essential to protect mothers from illegally abusing the drugs for the purpose of early pregnancy termination.

## Figures and Tables

**Table I:** Drug information.

Drug name	Medical use	Clinical dosage	FDA pregnancy category	Chemical structure
Isotretinoin (13-cis retinoic acid)	Acne	0.5–2 μg/kg/day	X	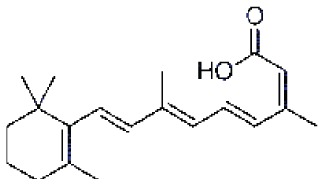
Misoprostol (prostaglandin E1 analog)	Gastric ulcers	0.4–0.8 μg/body/day	X	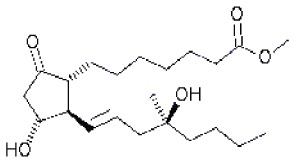
Methotrexate (amethopterin)	Cancer	5–10 μg/body/day	X	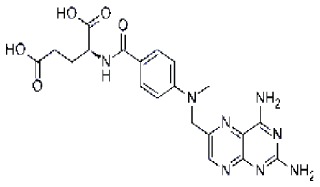
Mifepristone (RU-486)	Emergency contraceptive, abortion	200–600 μg/body/day	X	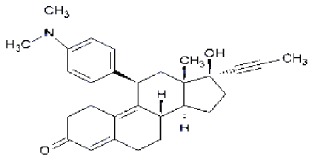
Levonorgestrel (l-norgestrel)	Contraception	1.5 μg/body/day	X	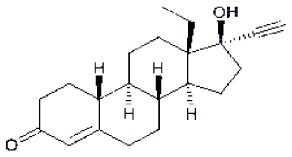

**Table II:** Hematological parameters following 3 days of oral treatment of the drugs in female mice (mean ± SD, n=3–5 mice).

		Isotretinoin (μg/kg/day)			Misoprostol (μg/kg/day)			Methotrexate (μg/kg/day)			Mifepristone (μg/kg/day)			Levonorgestrel (μg/kg/day)		
						
	Control	1	10	100	6.7	67	670	83	830	8300	3.3	33	330	25	250	2500
WBC (K/*μ*gl)	2.87	2.10	2.64	2.17	2.55	2.51	1.53	2.23	2.56	1.75	2.54	1.96	3.37	2.07	1.29	2.30
	±0.69	±1.00	±0.55	±1.02	±0.88	±0.79	±0.74^a^	±0.30	±1.34	±0.29	±0.45	±0.42	±0.89	±0.77	±0.50^a^	±1.39
LYM (K/*μ*gl)	1.60	1.72	2.08	1.82	2.02	1.62	1.14	1.69	1.91	1.18	2.05	1.52	2.91	1.71	0.92	1.75
	±0.55	±0.80	±0.60	±0.84	±1.06	±0.68	±0.45	±0.42	±1.10	±0.12	±0.49	±0.20	±0.76^a^	±0.64	±0.39	±1.08
MON (K/*μ*gl)	0.11	0.11	0.14	0.08	0.10	0.14	0.07	0.14	0.13	0.13	0.38	0.06	0.13	0.07	0.06	0.10
	±0.07	±0.02	±0.07	±0.05	±0.05	±0.05	±0.05	±0.09	±0.04	±0.03	±0.51	±0.04	±0.15	±0.04	±0.02	±0.07
GRA (K/*μ*gl)	1.17	0.28	0.43	0.27	0.43	0.76	0.33	0.40	0.53	0.44	0.36	0.37	0.33	0.29	0.32	0.46
	±0.48^b^	±0.21^b^	±0.15^b^	±0.16^b^	±0.30^b^	±0.18^a^	±0.34^b^	±0.22^b^	±0.26^b^	±0.19^b^	±0.31^b^	±0.23^b^	±0.32^b^	±0.15^b^	±0.19^b^	±0.28^b^
LY% (%)	55.38	82.43	78.03	84.25	75.43	53.03	77.40	75.05	70.45	68.30	80.95	79.00	85.57	83.03	70.68	75.03
	±15.65	±3.20^c^	±9.60^a^	±3.35^c^	±23.84^a^	±30.09	±14.35^a^	±11.07	±14.35	±9.40	±12.05^a^	±10.03^a^	±15.62^c^	±2.11^a^	±11.64	±8.16
MO% (%)	4.08	6.53	5.45	3.05	4.35	5.33	4.50	6.90	6.10	7.30	5.33	3.08	3.77	3.40	5.08	4.03
	±3.35	±4.70	±3.59	±1.62	±3.09	±1.20	±2.68	±5.06	±4.00	±1.31	±1.26	±1.36	±2.86	±1.57	±2.13	±0.74
GR% (%)	90.58	11.05	16.50	12.68	20.25	31.60	18.13	18.05	23.43	24.40	13.73	17.93	10.63	13.57	24.25	21.00
	±91.27^b^	±6.11^b^	±6.78^b^	±2.86^b^	±20.84^b^	±9.69^c^	±16.79^b^	±10.89^b^	±10.80^b^	±8.11^c^	±11.38^b^	±9.72^b^	±30.10^b^	±3.67^b^	±10.06^b^	±8.80^b^
RBC (M/*μ*gl)	8.72	7.62	8.65	8.26	8.06	9.13	9.14	8.77	7.97	8.14	9.33	9.08	9.41	8.63	7.07	9.28
	±0.48	±2.72	±0.44	±1.46	±0.52	±0.39	±0.29	±0.50	±1.61	±0.32	±0.79	±0.41	±1.64	±0.47	±2.67	±0.25
HGB (g/dl)	15.13	12.58	14.28	13.90	13.50	15.03	15.20	14.63	12.83	13.93	16.18	15.30	16.00	14.90	12.25	15.73
	±0.87	±5.25	±0.53	±2.95	±0.90	±0.52	±0.64	±0.80	±3.24	±0.47	±0.95	±0.66	±2.94	±0.36	±4.97	±0.55
HCT (%)	41.41	35.77	40.68	38.75	38.08	41.99	42.02	40.96	37.17	37.93	43.95	41.55	44.38	40.35	33.77	43.65
	±1.78	±12.90	±1.49	±7.17	±2.24	±0.94	±1.55	±1.62	±7.74	±1.98	±3.54	±1.86	±7.63^a^	±1.55	±12.84^a^	±0.96
MCV (fl)	47.50	46.75	47.25	46.75	47.25	46.00	45.75	46.50	46.50	46.67	47.00	46.00	47.00	46.67	47.50	47.00
	±1.29	±0.96	±0.96	±0.96	±0.96	±1.15	±0.50^a^	±1.29	±0.58	±0.58	±0.82	±0.82	±0.97	±1.15	±0.58	±1.00
MCH (pg)	17.35	16.00	16.55	16.75	16.75	16.48	16.63	16.68	15.93	17.13	17.35	16.88	17.03	17.23	17.05	16.93
	±0.47	±1.90^a^	±0.25^a^	±0.83	±0.31	±0.21	±0.31	±0.30	±1.07^a^	±0.06	±0.86	±0.19	±0.81	±0.80	±1.05	±0.71
MCHC (g/dl)	36.45	34.13	35.13	35.78	35.40	35.75	36.15	35.73	34.20	36.77	36.83	36.85	36.07	36.90	35.80	36.07
	±0.66	±3.76^a^	±0.62	±1.45	±0.50	±0.53	±0.37	±0.60	±2.12^a^	±0.67	±1.11	±0.21	±1.54	±1.15	±2.15	±1.06
PLT (K/*μ*gl)	465.00	574.75	579.00	382.00	456.25	490.75	583.00	710.00	409.67	400.50	533.75	571.25	650.33	596.33	380.75	667.33
	±53.20	±128.90	±180.30	±239.50	±174.70	±96.60	±25.20	±230.10	±172.30	±99.70	±82.90	±91.50	±177.30	±67.30	±216.60	±218.30

WBC, white blood cells; LYM, lymphocytes; MON, monocytes; GRA, granulocytes; LY%, percentage of lymphocytes; MO%, percentage of monocytes; GR%, percentage of granulocytes; RBC, red blood cells; HGB, hemoglobin; HCT, hematocrit; MCV, mean corpuscular volume; MCH, mean corpuscular hemoglobin; MCHC, mean corpuscular hemoglobin concentration; PLT, platelet.

a P<0.05,

b P<0.001 and

c P<0.01.

**Table III:** Clinical chemistry parameters following 3 days of oral treatment of the drugs in female mice (mean ± SD, n=3–5 mice).

		Isotretinoin (μg/kg/day)			Misoprostol (μg/kg/day)			Methotrexate (μg/kg/day)			Mifepristone (μg/kg/day)			Levonorgestrel (μg/kg/day)		
						
	Control	1	10	100	6.7	67	670	83	830	8300	3.3	33	330	25	250	2500
BUN (μg/Dl)	24.75	29.00	25.67	18.00	30.00	19.75	13.75	20.50	20.00	22.33	23.67	26.75	24.75	20.50	29.00	26.00
	±10.24	±8.72	±7.09	±4.55	±14.00	±3.50	±2.50	±4.20	±7.07	±5.69	±7.23	±3.77	±6.75	±9.19	±12.46	±4.00
CRSC (μg/Dl)	0.20	0.20	0.23	0.08	0.27	0.15	0.13	0.15	0.15	0.17	0.20	0.13	0.18	0.15	0.20	0.13
	±0.08	±0.08	±0.12^a^	±0.05	±0.12	±0.06	±0.05	±0.06	±0.07	±0.06	±0.00	±0.05	±0.10	±0.07	±0.00	±0.05
ALT (IU/l)	37.40	32.00	78.00	51.50	90.50	57.25	36.25	61.50	58.50	30.00	40.00	38.50	40.50	38.33	42.50	38.25
	±4.62	±6.38	±51.81	±32.35	±89.35^a^	±31.16	±13.40	±36.74	±40.25	±3.00	±4.32	±10.41	±15.07	±14.50	±23.90	±9.22
AST (IU/l)	98.25	98.25	230.75	178.50	159.00	139.25	102.25	143.50	258.50	96.67	116.25	106.00	156.00	162.67	134.25	112.75
	±18.63	±10.40	±185.91	±83.01	±79.28	±65.61	±20.01	±83.05	±300.88^a^	±10.50	±14.13	±22.05	±161.36	±105.08	±68.81	±31.13
ALP (IU/l)	76.80	91.25	144.00	116.00	97.00	123.50	144.00	113.00	97.75	89.00	107.00	118.00	81.50	76.33	124.50	93.00
	±15.34	±11.32	±54.36^b^	±15.38^a^	±6.83	±27.81^a^	±14.51^b^	±17.51	±48.94	±31.11	±26.99	±29.86^a^	9.19	54.88	23.81^a^	16.55
CK (IU/l)	101.25	92.33	208.00	272.00	176.00	178.25	87.50	69.50	100.00	432.00	420.00	136.50	508.75	541.00	477.75	202.00
	±87.52	±32.59	±158.04	±202.03	±128.31	±186.91	±36.13	±32.30	±45.21	±611.42	±660.05	±123.85	±819.60	±802.81	±462.31	±162.57
LDH (IU/l)	536.25	705.33	609.00	536.00	391.67	539.50	384.75	369.33	666.00	465.33	609.33	369.75	448.50	486.00	452.00	462.75
	±310.51	±158.86	±341.74	±175.92	±161.13	±122.24	±105.39	±203.64	±260.92	±131.35	±243.94^a^	±49.65	±161.60	±285.67	±137.25	±89.02
TBIL (μg/Dl)	0.09	0.10	0.11	0.06	0.06	0.09	0.06	0.08	0.10	0.05	0.07	0.05	-^nd^	0.05	0.05	0.04
	±0.02	±0.01	±0.06	±0.02^a^	±0.03	±0.02	±0.01	±0.01	±0.02	±0.00	±0.04	±0.02	-	±0.03^a^	±0.02^a^	±0.02
Tchol (μg/Dl)	94.60	93.25	102.50	95.25	78.25	88.50	93.25	97.75	109.25	90.33	90.25	127.00	158.33	93.00	105.00	109.25
	±22.61	±14.93	±28.62	±19.52	±7.23	±12.23	±21.41	±10.78	±8.92	±21.13	±8.18	±30.08^a^	±22.03^c^	±29.21	±38.22	±23.64
LIPA (IU/l)	26.00	23.00	23.00	24.00	43.00	23.25	22.00	28.33	28.75	36.67	32.00	24.25	29.25	32.67	27.75	23.50
	±7.48	±0.00	±5.00	±3.65	±15.12^c^	±2.75	±2.83	±6.66	±2.99	±8.33^a^	±11.34	±3.50	±6.18	±3.21	±0.96	±4.73
GLU (μg/Dl)	164.20	158.25	169.50	131.25	214.50	157.00	124.75	185.25	203.25	182.33	181.50	229.75	115.50	197.33	184.75	128.00
	±22.53	±30.21	±21.05	±6.02^a^	±32.42	±8.12	±23.19	±48.67	±40.73	±15.31	±21.52	±80.16	±7.59^a^	±11.72	±23.95	±27.78^a^
TP (g/Dl)	5.55	6.00	6.53	6.15	5.43	6.13	6.38	5.48	6.20	5.60	6.00	6.10	-	4.20	5.95	6.03
	±0.47	±0.22	±2.11	±0.19	±0.25	±0.29	±0.28	±0.40	±0.40	±0.85	±0.37	±0.34	-	±3.38	±0.44	±0.60
ALB (g/Dl)	3.85	3.95	3.40	3.73	3.67	3.63	3.90	3.63	3.70	3.50	3.88	3.60	3.75	4.05	3.65	3.97
	±0.57	±0.13	±0.72	±0.10	±0.21	±0.13	±0.16	±0.62	±0.28	±0.52	±0.43	±0.20	±0.26	±0.35	±0.17	0.21
Ca (μg/Dl)	9.75	11.00	11.85	11.15	11.38	11.05	11.18	11.73	11.28	11.30	10.95	10.98	14.07	11.60	11.45	12.63
	±3.00	±0.00	±2.22	±0.37	±0.82	±0.25	±0.42	±0.57	±0.91	±1.27	±1.04	±0.15	±3.02^c^	±0.70	±0.66	±1.47^c^
P (μg/Dl)	8.93	8.93	9.63	7.90	10.93	8.95	7.80	10.20	9.43	8.05	10.30	7.60	14.80	7.10	9.33	9.37
	±1.45	±1.11	±1.35	±0.65	±0.67	±0.89	±0.91	±1.28	±2.23	±0.35	±0.68	±0.24	±0.00	±6.09	±1.17	±2.63
UA (μg/Dl)	2.80	2.10	1.43	1.70	1.80	2.15	1.73	1.73	2.15	1.37	2.08	1.85	1.98	2.33	1.73	2.05
	±0.53	±0.00	±0.42^a^	±0.18^a^	±0.52	±0.74	±0.21^a^	±0.49	±1.18	±0.32^a^	±1.16	±0.47	±0.86	±1.10	±0.22^a^	±0.77

^nd^, not detectable. BUN, blood urea nitrogen; CRSC, creatinine; ALT, alanine aminotransferase; AST, aspartate aminotransferase; ALP, alkaline phosphatase; CK, creatine kinase; LDH, lactate dehydrogenase; TBIL, total bilirubin; Tchol, total cholesterol; LIPA, lipase; GLU, glucose; TP, total protein; ALB, albumin; Ca, calcium; P, inorganic phosphorus; UA, uric acid.

a P<0.05,

b P<0.01 and

c P<0.001.

**Table IV:** Gross necropsy findings following 3 days of oral treatment of the drugs in female mice.

		Isotretinoin (mg/kg/day)			Misoprostol (μg/kg/day)			Methotrexate (μg/kg/day)			Mifepristone (mg/kg/day)			Levonorgestrel (μg/kg/day)		
						
Finding	Control	1	10	100	6.7	67	670	83	830	8300	3.3	33	330	25	250	2500
Number/group	5	4	4	4	4	4	4	4	4	4	4	4	4	4	4	4
Mortality	0	0	0	0	0	0	0	0	0	1	0	0	0	0	0	0
Uterus enlargement	0	0	0	0	0	0	0	0	0	0	0	0	2	0	2	1
Ovarian dropsy	0	0	0	0	0	0	0	0	0	0	0	0	2	0	3	0
Intestine congestion	0	0	0	0	0	0	0	0	2	4	0	0	0	0	0	1
